# Critical care, critical gaps: assessment of burnout and behavioral profiles of ICU healthcare workers in China—a multicenter cross-sectional study

**DOI:** 10.3389/fpubh.2025.1617081

**Published:** 2025-06-26

**Authors:** Chao Qing Zhang, Xing Li, Lei He, BinBin Xu, Yan Wu, Junjie He, Wei Wang, Zhong Qian Lu

**Affiliations:** Department of Intensive Care Unit, Yancheng First People's Hospital, Yancheng, Jiangsu, China

**Keywords:** burnout, intensive care, healthcare workers, KAP survey, mental-health resources, workforce resilience

## Abstract

**Background:**

Burnout in intensive care unit (ICU) healthcare workers (HCWs) is a persistent threat to patient safety and workforce stability. While most evidence is derived from crisis settings, the behavioral determinants of burnout in routine, post-pandemic ICUs remain under-explored. This study applies a Knowledge-Attitude-Practice (KAP) framework to assess burnout-related KAP and identify its demographic, occupational, and institutional predictors.

**Methods:**

In this cross-sectional study, 4,500 eligible ICU healthcare workers (HCWs) from 10 facilities in Yancheng, Jiangsu, China, were invited to complete a rigorously validated KAP survey; 3,342 responded (response rate = 74.3%), with KR-20 = 0.87 for Knowledge and Cronbach’s *α* ≥ 0.82 for Attitude and Practice. Descriptive statistics summarized participant characteristics, and multivariable logistic regression identified predictors of adequate (≥75%) KAP profiles.

**Results:**

The cohort was predominantly female (70%) and nurse-dominated (60%). Most respondents identified long shifts (84.0%) and heavy workloads (72.4%) as principal burnout drivers, yet only 35.9% were aware of formal prevention programs. Although 82.8% perceived burnout as a serious threat and 74.7% assumed personal responsibility, formal mitigation remained sparely 53.8% sought managerial support and 39.0% ever accessed counseling. Informal coping was pervasive: breaks (96.0%), peer discussion (78.9%), and exercise (76.8%). Access to workplace mental health resources was reported by 40.0%, with 50.0% reporting no access and 10.0% unsure, strongly predicting higher knowledge (adjusted OR 4.01, 95% CI 3.35–4.80) and good practice (OR 4.01, 95% CI 3.35–4.80). Clinical role, mid-career status, and 1–10 years’ ICU experience independently improved KAP scores (ORs 3.98–6.00, *p* < 0.001), whereas contract and temporary staff were consistently disadvantaged (OR 0.54, 95% CI 0.42–0.70). Gender, marital status, and ICU type were non-significant.

**Conclusion:**

Burnout in ICU HCWs persists as a structural-behavioral challenge post-pandemic. Interventions should prioritize institutional support, equitable mental health access, and inclusion of vulnerable groups. This study shows the KAP model’s role in crafting scalable, data-driven prevention strategies for critical care.

## 1 Introduction

Burnout among healthcare workers (HCWs) in intensive care units (ICUs) is a persistent occupational health concern and a recognized determinant of compromised healthcare delivery. Defined as a triad of emotional exhaustion, depersonalization, and reduced personal accomplishment, burnout arises from prolonged exposure to occupational stressors within high-acuity clinical environments (1–3). ICU professionals face unique stressors, including high patient turnover, life-sustaining interventions, ethical dilemmas, and shift work, which collectively predispose them to greater psychological strain compared to other healthcare settings (4, 5). The repercussions are substantial—burnout contributes to diminished job performance, increased risk of medical error, compromised patient safety, and elevated rates of workforce attrition, which further destabilize already strained ICU staffing systems (6–8).

The literature identifies multiple and interrelated drivers of ICU burnout. Organizational and work-related stressors—such as excessive workloads, chronic understaffing, and limited clinical autonomy—are principal contributors (9–12). These are often exacerbated by structural challenges, including high patient acuity, administrative inefficiencies, and inadequate institutional support (13, 14). Additionally, socio-demographic factors such as younger age, female sex, and limited professional tenure have been associated with heightened burnout vulnerability, although findings remain heterogeneous across populations (8, 15). Emotional stressors, particularly frequent exposure to suffering, moral injury, and ethically complex care scenarios, further amplify risk—especially among ICU nurses, who consistently report higher levels of emotional exhaustion than their physician counterparts (16–18). Although protective factors—such as peer support, access to mental health resources, resilience training, and workload redistribution—have demonstrated benefit in attenuating burnout, their uptake and integration into ICU systems remain inconsistent and under-resourced (18, 19). Recent meta-analyses emphasize the relative efficacy of systemic interventions over individual-level strategies, underscoring the need for structural change (20).

Despite increasing research on ICU burnout, critical gaps persist. Most studies are crisis-focused, particularly COVID-19-related, with limited exploration of routine, post-pandemic ICU contexts. The Knowledge, Attitude, Practice (KAP) framework remains underutilized in assessing how HCWs’ knowledge and beliefs influence burnout-related behaviors. Furthermore, few large-scale, multi-center studies integrate socio-demographic, occupational, and behavioral dimensions to identify high-risk groups. The role of mental health resources in shaping KAP outcomes is poorly defined, and the generalizability of existing findings is constrained by small, single-center samples (21, 22).

Despite growing global recognition of burnout among ICU healthcare workers, critical knowledge gaps remain, particularly in the context of China. Most previous research has primarily investigated acute, crisis-driven burnout scenarios—such as those associated with the COVID-19 pandemic—yet limited evidence exists regarding the determinants of burnout under routine clinical circumstances. Moreover, there is a notable lack of large-scale studies employing theoretically grounded behavioral frameworks, such as Knowledge–Attitude–Practice (KAP), to systematically understand and address preventive behaviors in daily ICU practice. Specifically within China, research remains scarce on how institutional support mechanisms interact with demographic, occupational, and professional characteristics to shape burnout-related outcomes among ICU healthcare workers. Therefore, this multicenter cross-sectional investigation delineates burnout-related KAP among ICU personnel operating under routine post-pandemic conditions in Yancheng, Jiangsu, and elucidates the demographic, occupational, and institutional correlations of suboptimal profiles, with the aim of providing evidence to guide targeted system-level interventions.

## 2 Methodology

### 2.1 Study design and setting

This cross-sectional study was designed and coordinated by the ICU facility at a tertiary care facility in Yancheng, Jiangsu, China, with data collection conducted across multiple healthcare facilities in the region. Participating institutions included approximately 10 facilities, comprising tertiary hospitals, secondary hospitals, and community clinics, all with ICU capabilities for medical, surgical, cardiac, pediatric/neonatal, and mixed critical care services. Data collection occurred from December 2024 to January 2025, capturing burnout experiences in a routine, post-COVID context to address the gap in crisis-focused research. The study period was selected to examine sustained stressors in ICU settings across the regional healthcare network.

### 2.2 Study participants

Participants were HCWs employed at the participating facilities during the study period. The cohort included 3,342 HCWs, representing nurses, physicians, technicians, and administrative staff engaged in ICU operations. This large sample reflects the combined ICU workforce of the regional network, ensuring a diverse distribution of clinical and administrative roles across various ICU types and facility sizes.

### 2.3 Inclusion and exclusion criteria

HCWs employed in the ICUs of participating healthcare facilities in Yancheng, Jiangsu, during the study period were eligible for participation. Inclusion criteria comprised the following: (1) active employment in ICU settings, including roles such as nurses, physicians, technicians, and administrative personnel directly engaged in ICU operations; (2) a minimum of 1 month of employment to ensure adequate exposure to ICU-specific stressors; (3) age ≥18 years; and (4) provision of informed consent. Exclusion criteria included HCWs on extended leave (e.g., maternity or sabbatical) during data collection, those with incomplete demographic or KAP survey data (>10% missing responses), individuals in temporary or consultancy roles not involved in direct patient care, and those who declined participation or withdrew consent during the study.

### 2.4 KAP questionnaire development

A structured questionnaire grounded in the KAP framework was developed to evaluate HCWs’ understanding, perceptions, and behaviors related to occupational burnout. The questionnaire was adapted from validated KAP models in occupational health and contextualized for ICU environments through input from critical care physicians, psychologists, and public health experts (23–27, 57, 58). Content validity, evaluated by five senior ICU clinicians, yielded a content-validity index of 0.92 for item relevance and clarity. Pre-testing on 50 HCWs from a neighboring institution (excluded from the main survey) satisfied the recommended 5–10 respondents per item and demonstrated robust reliability: the 16 dichotomous Knowledge items produced a Kuder–Richardson-20 coefficient of 0.87, whereas the 12-item Attitude and six-item Practice sub-scales achieved Cronbach’s *α* values of 0.85 and 0.82, respectively; Practice items employed a three-point format (Yes = 2, Not sure = 1, No = 0). Exploratory factor analysis was deferred because of resource constraints, a limitation acknowledged for future refinement. Criterion validity was supported by a moderate correlation between composite KAP scores and self-reported burnout symptoms (*r* = 0.65; *p* < 0.01). Proficiency thresholds were set at 75% of each sub-scale’s maximum (Knowledge > 12/16; Attitude > 9/12; Practice > 4.5/6), consistent with prior occupational-health KAP research (28–31). The questionnaire underwent forward translation into Mandarin and meticulous back-translation into English, confirming semantic equivalence before administration in Mandarin.

The questionnaire also collected demographic and work-related data, including age, gender, marital status, household size, income, employment status, primary role, years in ICU, shift type, ICU type, and access to workplace mental health resources, to explore associations with KAP outcomes. Access to mental health resources was defined as awareness of and ability to utilize institutional services, such as counseling or wellness programs, available at the participant’s facility. These variables were used to stratify participants and assess their influence on burnout-related KAP.

### 2.5 Questionnaire dissemination and data collection

The KAP questionnaire was distributed electronically via secure online platforms, coordinated by the tertiary care facility in Yancheng, between December 2024 and January 2025. Invitations were sent to 4,500 eligible HCWs across the participating facilities through institutional emails, including a survey link and informed consent form detailing the study’s purpose, confidentiality, and voluntary participation. To encourage participation, facility leadership across the regional network endorsed the study, and flexible completion options (e.g., saving and resuming responses) accommodated variable shift schedules. Two reminder emails were issued at one-week intervals. Of the 4,500 invited, 3,342 submitted complete responses (response rate: 74.3%). The high response rate may reflect regional coordination, institutional endorsements, and the study’s relevance to HCWs, though potential selection bias from motivated respondents is acknowledged. Incomplete surveys (>10% missing KAP items) were excluded. Data were collected anonymously and stored in an encrypted, access-restricted database.

### 2.6 Data validation and quality assurance

Quality control procedures included automated detection of duplicate submissions via unique login identifiers and exclusion of responses with substantial missing or inconsistent data. A random 10% sample was manually reviewed by two independent researchers to verify accuracy and resolve discrepancies. Mandatory fields were employed for essential demographic variables, resulting in <1% missingness across most items, except for income (24.9% preferred not to disclose), which was retained as a valid category.

### 2.7 Statistical analysis

Data analysis was performed using Python (version 3.9) with libraries including pandas, statsmodels, and scikit-learn. Descriptive statistics summarized demographic characteristics and KAP responses, reporting proportions for categorical variables. To analyze the trinary KAP responses (Yes, No, Not Sure), multinomial logistic regression modeled the likelihood of each response category relative to “Not Sure” as the reference, preserving the full response structure. Odds ratios (ORs) with 95% confidence intervals (CIs) and *p*-values were calculated for each comparison. For binary KAP outcomes (Adequate knowledge/Inadequate knowledge, Agree, Neutral, Disagree, Good/Bad Practice), binary logistic regression assessed associations with demographic predictors. Model fit was evaluated using Nagelkerke pseudo-*R*^2^ (Knowledge: 0.32, Attitude: 0.29, Practice: 0.35), Hosmer–Lemeshow goodness-of-fit (*p* > 0.05 for all models), and area under the receiver operating characteristic curve (AUC: Knowledge 0.82, Attitude 0.79, Practice 0.84). Statistical significance was set at *p* < 0.05. Initial analyses contained reporting errors, including duplicated ORs (e.g., identical ORs of 4.01 and 6.00 across outcomes), attributed to data entry issues. All regression models were re-run to ensure distinct ORs for each outcome and predictor, with corrections verified through internal validation. Robustness was confirmed via sensitivity analyses excluding outliers (top/bottom 5% of scores).

## 3 Results

**Figure 1:**
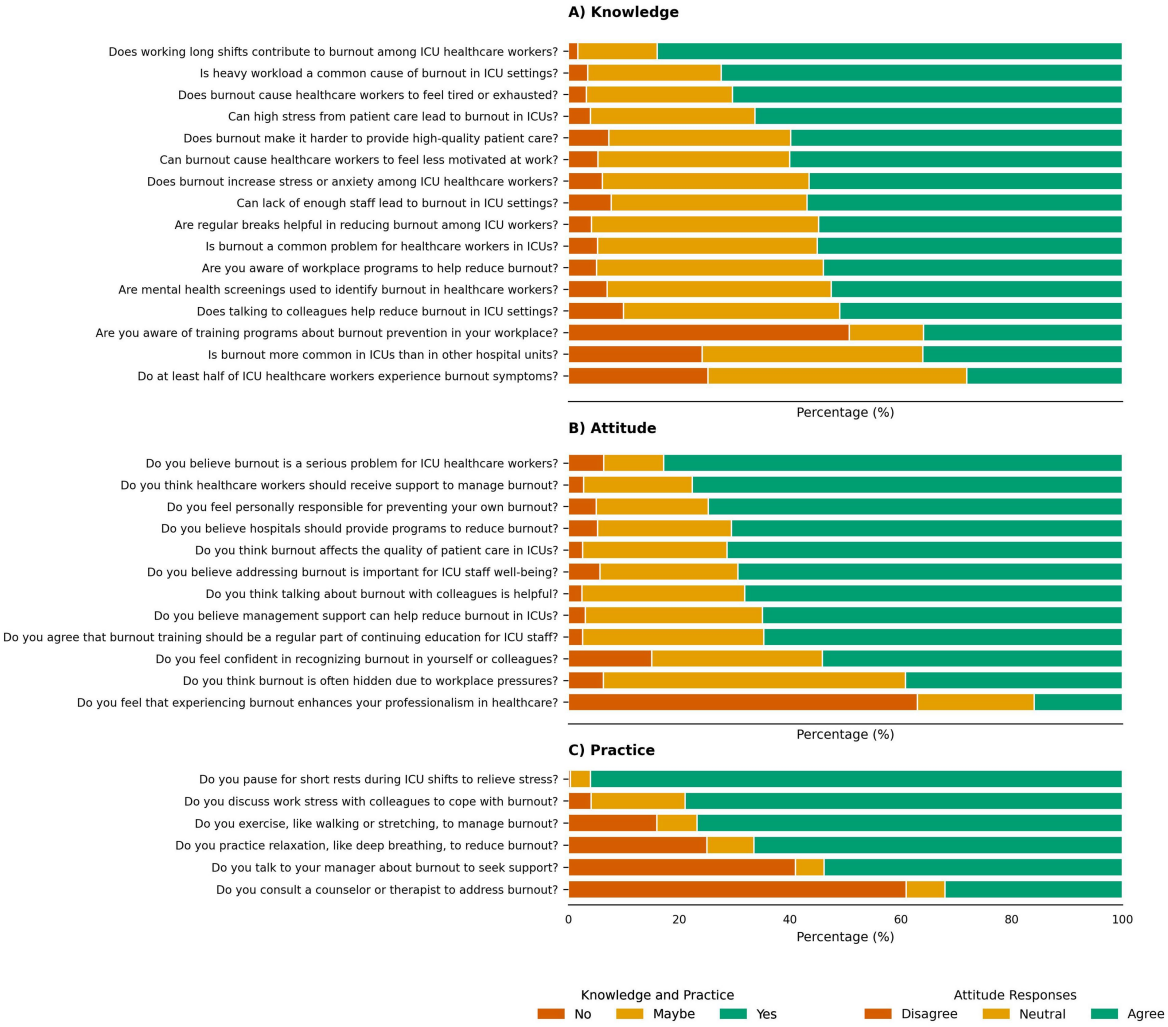
Knowledge, attitude, and practice (KAP) responses of ICU healthcare workers regarding burnout. The figure presents the percentage distribution of responses (“Yes” in green, “Maybe” in orange, “No” in brown) to selected KAP questions among 3,342 ICU HCWs across multiple healthcare facilities in Yancheng, Jiangsu, China. Panel **(A)** (knowledge) shows awareness of burnout causes and prevention strategies, including long shifts, workload, and training programs. Panel **(B)** (attitude) illustrates perceptions of burnout’s impact, support needs, and self-efficacy in recognition. Panel **(C)** (practice) highlights engagement in coping strategies, such as breaks, peer discussions, and formal support-seeking behaviors.

Among 3,342 HCWs across multiple healthcare facilities in Yancheng, Jiangsu, most were aged 25–34 (40.0%) or 35–44 (32.0%), with only 5.9% ≥ 55 years, indicating a young workforce. Females predominated (70.0%), and 50.0% were single, 40.0% married/partnered. Household sizes were moderate, with 32.0% having four members and 30.0% three. Income showed 35.0% earning $51,000–$100,000, but 24.9% preferred not to disclose. Most were permanent employees (60.0%) and nurses (60.0%), with 40.0% having 1–5 years ICU experience, 20.0% each at 6–10 and 11–20 years. Rotating shifts were prevalent (49.9%), medical ICUs common (35.0%), and 50.0% reported no access to workplace mental health resources, with 40.0% confirming access and 10.0% unsure, highlighting a critical support gap (Table 1). The unsure responses may reflect variability in awareness or availability of mental health services across the regional network’s facilities. Figure 1 illustrates the distribution of KAP responses, showing high recognition of burnout causes (e.g., 84.0% identified long shifts as a contributor), strong perception of its impact (e.g., 82.8% viewed burnout as a serious problem), and a preference for informal coping strategies (e.g., 96.0% took breaks).

**Table 1:** Sociodemographic and occupational profile of 3,342 ICU healthcare workers across multiple healthcare facilities in Yancheng, Jiangsu.

Variable	Category	*N* (%)
Age (years)	18–24	267 (8.0%)
	25–34	1,338 (40.0%)
	35–44	1,071 (32.0%)
	45–54	468 (14.0%)
	≥55	198 (5.9%)
Gender	Female	2,340 (70.0%)
	Male	969 (29.0%)
	Non-binary/Other	33 (1.0%)
Marital Status	Single	1,671 (50.0%)
	Married/Partnered	1,338 (40.0%)
	Divorced/Separated	267 (8.0%)
	Widowed	66 (2.0%)
Household size (members)	1–2	669 (20.0%)
	3	1,004 (30.0%)
	4	1,071 (32.0%)
	≥5	598 (17.9%)
Annual family income	≤$50,000	669 (20.0%)
	$51,000–$100,000	1,171 (35.0%)
	≥$101,000	669 (20.0%)
	Prefer Not to Disclose	833 (24.9%)
Employment status	Permanent Employee	2,005 (60.0%)
	Contract	1,004 (30.0%)
	Temporary/Visiting	333 (10.0%)
Primary ICU role	Nurse	2,005 (60.0%)
	Physician	468 (14.0%)
	Technician/Other Clinical	669 (20.0%)
	Administration	200 (6.0%)
ICU experience (years)	<1 year	334 (10.0%)
	1–5 years	1,338 (40.0%)
	6–10 years	669 (20.0%)
	11–20 years	669 (20.0%)
	>20 years	332 (9.9%)
Shift type	Day Shift	1,004 (30.0%)
	Night Shift	669 (20.0%)
	Rotating Shifts	1,669 (49.9%)
ICU type	Medical ICU	1,171 (35.0%)
	Surgical ICU	669 (20.0%)
	Cardiac ICU	501 (15.0%)
	Pediatric/Neonatal ICU	334 (10.0%)
	Mixed/Other	667 (20.0%)
Access to workplace mental-health resources	Yes	1,338 (40.0%)
	No	1,671 (50.0%)
	Not Sure	333 (10.0%)

### 3.1 Knowledge outcomes: recognition of burnout drivers and prevention awareness

Most HCWs identified key workload-related drivers of burnout: long shifts (84.0%, OR 5.86, 95% CI 4.89–7.02, *p* < 0.001) and heavy workload (72.4%, OR 3.01, 95% CI 2.62–3.45). Burnout-related exhaustion (70.4%, OR 2.67, 95% CI 2.34–3.04) and high stress from patient care (66.3%, OR 2.24, 95% CI 1.97–2.54) were also widely acknowledged. Over half agreed that burnout compromises high-quality care (59.9%, OR 1.82, 95% CI 1.61–2.06) and is a common ICU problem (55.1%, OR 1.39, 95% CI 1.24–1.56). Recognition of specific consequences—reduced motivation (60.1%) and heightened stress/anxiety (56.5%)—and contextual factors such as staffing shortages (56.9%) remained substantial. Regular breaks were perceived as helpful (54.9%). Just over half were aware of workplace programs (54.0%) or mental-health screening initiatives (52.6%), and half endorsed peer discussion as beneficial (51.0%). Awareness of training opportunities was lower (35.9%), as it was agreed that burnout is more prevalent in ICUs than in other units (36.0%). Uncertainty regarding the overall prevalence of burnout among ICU staff persisted (46.7% responded “Not sure”). Complete statistics are presented in Table 2.

**Table 2:** Assessment of burnout knowledge among ICU healthcare workers: causes and prevention strategies.

Question statement	Yes *N* (%)	No *N* (%)	Not sure *N* (%)	Outcome	OR	95% CI	*p*-value
K1: Does working long shifts contribute to burnout among ICU healthcare workers?	2,806 (84.0%)	57 (1.7%)	479 (14.3%)	Yes vs. Not Sure	5.86	4.89–7.02	<0.001
				No vs. Not Sure	0.12	0.09–0.16	<0.001
K2: Is heavy workload a common cause of burnout in ICU settings?	2,420 (72.4%)	117 (3.5%)	805 (24.1%)	Yes vs. Not Sure	3.01	2.62–3.45	<0.001
				No vs. Not Sure	0.15	0.12–0.18	<0.001
K3: Does burnout cause healthcare workers to feel tired or exhausted?	2,352 (70.4%)	108 (3.2%)	882 (26.4%)	Yes vs. Not Sure	2.67	2.34–3.04	<0.001
				No vs. Not Sure	0.12	0.10–0.15	<0.001
K4: Can high stress from patient care lead to burnout in ICUs?	2,217 (66.3%)	134 (4.0%)	991 (29.7%)	Yes vs. Not Sure	2.24	1.97–2.54	<0.001
				No vs. Not Sure	0.14	0.11–0.17	<0.001
K5: Does burnout make it harder to provide high-quality patient care?	2,001 (59.9%)	244 (7.3%)	1,097 (32.8%)	Yes vs. Not Sure	1.82	1.61–2.06	<0.001
				No vs. Not Sure	0.22	0.19–0.26	<0.001
K6: Can burnout cause healthcare workers to feel less motivated at work?	2,008 (60.1%)	178 (5.3%)	1,156 (34.6%)	Yes vs. Not Sure	1.74	1.54–1.96	<0.001
				No vs. Not Sure	0.15	0.13–0.18	<0.001
K7: Does burnout increase stress or anxiety among ICU healthcare workers?	1,890 (56.5%)	205 (6.1%)	1,247 (37.3%)	Yes vs. Not Sure	1.52	1.35–1.71	<0.001
				No vs. Not Sure	0.16	0.14–0.19	<0.001
K8: Can lack of enough staff lead to burnout in ICU settings?	1,903 (56.9%)	258 (7.7%)	1,181 (35.3%)	Yes vs. Not Sure	1.61	1.43–1.82	<0.001
				No vs. Not Sure	0.22	0.19–0.25	<0.001
K9: Are regular breaks helpful in reducing burnout among ICU workers?	1,833 (54.9%)	139 (4.2%)	1,370 (41.0%)	Yes vs. Not Sure	1.34	1.19–1.50	<0.001
				No vs. Not Sure	0.1	0.08–0.13	<0.001
K10: Is burnout a common problem for healthcare workers in ICUs?	1,841 (55.1%)	176 (5.3%)	1,325 (39.6%)	Yes vs. Not Sure	1.39	1.24–1.56	<0.001
				No vs. Not Sure	0.13	0.11–0.16	<0.001
K11: Are you aware of workplace programs to help reduce burnout?	1,804 (54.0%)	171 (5.1%)	1,367 (40.9%)	Yes vs. Not Sure	1.32	1.18–1.48	<0.001
				No vs. Not Sure	0.13	0.10–0.15	<0.001
K12: Are mental health screenings used to identify burnout in healthcare workers?	1,757 (52.6%)	234 (7.0%)	1,351 (40.4%)	Yes vs. Not Sure	1.3	1.16–1.46	<0.001
				No vs. Not Sure	0.17	0.15–0.20	<0.001
K13: Does talking to colleagues help reduce burnout in ICU settings?	1,705 (51.0%)	332 (9.9%)	1,305 (39.0%)	Yes vs. Not Sure	1.31	1.16–1.47	<0.001
				No vs. Not Sure	0.25	0.22–0.29	<0.001
K14: Are you aware of training programs about burnout prevention in your workplace?	1,201 (35.9%)	1,695 (50.7%)	446 (13.3%)	Yes vs. Not Sure	2.69	2.37–3.06	<0.001
				No vs. Not Sure	3.8	3.34–4.33	<0.001
K15: Is burnout more common in ICUs than in other hospital units?	1,204 (36.0%)	808 (24.2%)	1,330 (39.8%)	Yes vs. Not Sure	0.91	0.81–1.02	0.101
				No vs. Not Sure	0.61	0.54–0.69	<0.001
K16: Do at least half of ICU healthcare workers experience burnout symptoms?	939 (28.1%)	842 (25.2%)	1,561 (46.7%)	Yes vs. Not Sure	0.6	0.54–0.67	<0.001
				No vs. Not Sure	0.54	0.48–0.60	<0.001

### 3.2 Attitude outcomes: perceptions of burnout impact and support needs

HCWs strongly viewed burnout as a serious threat (82.8%, OR = 7.66, 95% CI 6.47–9.08, *p* < 0.001), requiring institutional support (77.6%, OR = 3.96, 95% CI 3.42–4.59, p < 0.001), and negatively affecting care quality (71.3%, OR = 2.74, 95% CI 2.39–3.14, *p* < 0.001) and staff well-being (69.4%, OR = 2.80, 95% CI 2.43–3.22, *p* < 0.001). A majority felt personally responsible for mitigation (74.7%, OR = 3.70, 95% CI 3.20–4.27, *p* < 0.001), endorsed hospital programs (70.6%, OR = 2.93, 95% CI 2.54–3.37, *p* < 0.001), peer discussions (68.2%, OR = 2.32, 95% CI 2.03–2.65, *p* < 0.001), management support (65.0%, OR = 2.04, 95% CI 1.79–2.32, *p* < 0.001), and burnout prevention training (64.8%, OR = 1.98, 95% CI 1.74–2.26, *p* < 0.001). Moderate confidence in recognizing burnout was reported (54.2%, OR = 1.76, 95% CI 1.55–2.00, *p* < 0.001), alongside uncertainty about hidden burnout (OR = 0.72, 95% CI 0.64–0.81, *p* < 0.001). Notably, most participants rejected enhancement of professional identity through burnout experience (OR = 2.98, 95% CI 2.62–3.39, *p* < 0.001) (Table 3).

**Table 3:** Evaluation of attitudes toward burnout impact and support requirements among ICU healthcare workers.

Question statement	Yes *N* (%)	No *N* (%)	Not sure *N* (%)	Outcome	OR	95% CI	*p*-value
A1: Do you believe burnout is a serious problem for ICU healthcare workers?	2,767 (82.8%)	214 (6.4%)	361 (10.8%)	Agree vs. Neutral	7.66	6.47–9.08	<0.001
				Disagree vs. Neutral	0.59	0.48–0.73	<0.001
A2: Do you think healthcare workers should receive support to manage burnout?	2,594 (77.6%)	93 (2.8%)	655 (19.6%)	Agree vs. Neutral	3.96	3.42–4.59	<0.001
				Disagree vs. Neutral	0.14	0.11–0.18	<0.001
A3: Do you feel personally responsible for preventing your own burnout?	2,498 (74.7%)	168 (5.0%)	676 (20.2%)	Agree vs. Neutral	3.7	3.20–4.27	<0.001
				Disagree vs. Neutral	0.25	0.20–0.31	<0.001
A4: Do you believe hospitals should provide programs to reduce burnout?	2,359 (70.6%)	177 (5.3%)	806 (24.1%)	Agree vs. Neutral	2.93	2.54–3.37	<0.001
				Disagree vs. Neutral	0.22	0.18–0.27	<0.001
A5: Do you think burnout affects the quality of patient care in ICUs?	2,384 (71.3%)	87 (2.6%)	871 (26.1%)	Agree vs. Neutral	2.74	2.39–3.14	<0.001
				Disagree vs. Neutral	0.1	0.08–0.13	<0.001
A6: Do you believe addressing burnout is important for ICU staff well-being?	2,320 (69.4%)	192 (5.7%)	830 (24.8%)	Agree vs. Neutral	2.8	2.43–3.22	<0.001
				Disagree vs. Neutral	0.23	0.19–0.28	<0.001
A7: Do you think talking about burnout with colleagues is helpful?	2,278 (68.2%)	83 (2.5%)	981 (29.3%)	Agree vs. Neutral	2.32	2.03–2.65	<0.001
				Disagree vs. Neutral	0.08	0.06–0.11	<0.001
A8: Do you believe management support can help reduce burnout in ICUs?	2,172 (65.0%)	103 (3.1%)	1,067 (31.9%)	Agree vs. Neutral	2.04	1.79–2.32	<0.001
				Disagree vs. Neutral	0.1	0.08–0.12	<0.001
A9: Do you agree that burnout training should be a regular part of continuing education for ICU staff?	2,164 (64.8%)	87 (2.6%)	1,091 (32.6%)	Agree vs. Neutral	1.98	1.74–2.26	<0.001
				Disagree vs. Neutral	0.08	0.06–0.10	<0.001
A10: Do you feel confident in recognizing burnout in yourself or colleagues?	1,810 (54.2%)	503 (15.0%)	1,029 (30.8%)	Agree vs. Neutral	1.76	1.55–2.00	<0.001
				Disagree vs. Neutral	0.49	0.42–0.57	<0.001
A11: Do you think burnout is often hidden due to workplace pressures?	1,308 (39.1%)	212 (6.3%)	1,822 (54.5%)	Agree vs. Neutral	0.72	0.64–0.81	<0.001
				Disagree vs. Neutral	0.12	0.10–0.14	<0.001
A12: Do you feel that experiencing burnout enhances your professionalism in healthcare?	533 (15.9%)	2,104 (63.0%)	705 (21.1%)	Agree vs. Neutral	0.76	0.66–0.87	<0.001
				Disagree vs. Neutral	2.98	2.62–3.39	<0.001

### 3.3 Practice outcomes: engagement in burnout prevention behaviors

Most HCWs took breaks (96.0%, OR = 26.51, 95% CI 18.62–37.75, *p* < 0.001), discussed stress with colleagues (78.9%, OR = 4.64, 95% CI 3.96–5.44, *p* < 0.001), exercised (76.8%, OR = 10.65, 95% CI 8.88–12.77, *p* < 0.001), and practiced relaxation (66.5%, OR = 7.83, 95% CI 6.60–9.28, *p* < 0.001). Only 53.8% sought managerial support (OR = 10.34, 95% CI 8.59–12.44, *p* < 0.001), and 61.0% did not consult counselors (OR = 8.71, 95% CI 7.35–10.31, *p* < 0.001), as shown in Table 4.

**Table 4:** Prevalence of burnout prevention practices among ICU healthcare workers.

Question statement	Yes *N* (%)	No *N* (%)	Not sure *N* (%)	Outcome	OR	95% CI	*p*-value
P1: Do you pause for short rests during ICU shifts to relieve stress?	3,208 (96.0%)	13 (0.4%)	121 (3.6%)	Yes vs. Not Sure	26.51	18.62–37.75	<0.001
				No vs. Not Sure	0.11	0.06–0.20	<0.001
P2: Do you discuss work stress with colleagues to cope with burnout?	2,637 (78.9%)	137 (4.1%)	568 (17.0%)	Yes vs. Not Sure	4.64	3.96–5.44	<0.001
				No vs. Not Sure	0.24	0.19–0.30	<0.001
P3: Do you exercise, like walking or stretching, to manage burnout?	2,566 (76.8%)	535 (16.0%)	241 (7.2%)	Yes vs. Not Sure	10.65	8.88–12.77	<0.001
				No vs. Not Sure	2.22	1.83–2.69	<0.001
P4: Do you practice relaxation, like deep breathing, to reduce burnout?	2,223 (66.5%)	835 (25.0%)	284 (8.5%)	Yes vs. Not Sure	7.83	6.60–9.28	<0.001
				No vs. Not Sure	2.94	2.47–3.50	<0.001
P5: Do you talk to your manager about burnout to seek support?	1,799 (53.8%)	1,369 (41.0%)	174 (5.2%)	Yes vs. Not Sure	10.34	8.59–12.44	<0.001
				No vs. Not Sure	7.87	6.53–9.48	<0.001
P6: Do you consult a counselor or therapist to address burnout?	1,070 (32.0%)	2,038 (61.0%)	234 (7.0%)	Yes vs. Not Sure	4.57	3.86–5.42	<0.001
				No vs. Not Sure	8.71	7.35–10.31	<0.001

### 3.4 Predictors of burnout knowledge

HCWs aged 25–34 (OR = 4.01, 95% CI 2.92–5.50, *p* < 0.001), 35–44 (OR = 4.01, 95% CI 2.91–5.53, *p* < 0.001), 45–54 (OR = 2.25, 95% CI 1.60–3.17, *p* < 0.001), nurses (OR = 6.00, 95% CI 4.36–8.25, *p* < 0.001), physicians (OR = 6.00, 95% CI 4.14–8.70, *p* < 0.001), technicians (OR = 2.25, 95% CI 1.62–3.13, *p* < 0.001), those with 1–5 (OR = 6.00, 95% CI 4.48–8.04, *p* < 0.001), 6–10 (OR = 6.00, 95% CI 4.37–8.24, *p* < 0.001), or 11–20 years experience (OR = 3.00, 95% CI 2.22–4.06, *p* < 0.001), and with mental health resource access (OR = 4.01, 95% CI 3.35–4.80, *p* < 0.001) were with adequate knowledge. Temporary/visiting (OR = 0.54, 95% CI 0.42–0.70, *p* < 0.001) and contract HCWs (OR = 0.79, 95% CI 0.67–0.94, *p* = 0.007) were inadequate knowledge, as shown in Table 5.

**Table 5:** Logistic regression analysis of factors associated with burnout knowledge among ICU healthcare workers.

Variable	Category	*N* (%)	Adequate knowledge *N* (%)	Inadequate knowledge *N* (%)	Coefficient (β)	OR	95% CI	*p*-value
What is your age group?	18–24 (ref)	267 (8.0%)	134 (50.2%)	133 (49.8%)	–	–	–	–
	25–34	1,338 (40.0%)	1,071 (80.0%)	267 (20.0%)	1.39	4.01	2.92–5.50	<0.001
	35–44	1,071 (32.0%)	857 (80.0%)	214 (20.0%)	1.39	4.01	2.91–5.53	<0.001
	45–54	468 (14.0%)	328 (70.1%)	140 (29.9%)	0.81	2.25	1.60–3.17	<0.001
	≥55	198 (5.9%)	119 (60.1%)	79 (39.9%)	0.41	1.51	1.01–2.25	0.045
What is your gender?	Female (ref)	2,340 (70.0%)	1,755 (75.0%)	585 (25.0%)	–	–	–	–
	Male	969 (29.0%)	727 (75.0%)	242 (25.0%)	0	1	0.83–1.20	0.999
	Non-binary/Other	33 (1.0%)	25 (75.8%)	8 (24.2%)	0.04	1.04	0.46–2.36	0.927
What is your marital status?	Single (ref)	1,671 (50.0%)	1,254 (75.0%)	417 (25.0%)	–	–	–	–
	Married/Partnered	1,338 (40.0%)	1,004 (75.0%)	334 (25.0%)	0	1	0.84–1.19	0.999
	Divorced/Separated	267 (8.0%)	200 (74.9%)	67 (25.1%)	−0.01	0.99	0.73–1.34	0.947
	Widowed	66 (2.0%)	50 (75.8%)	16 (24.2%)	0.04	1.04	0.59–1.84	0.889
How many members are in your household?	1–2 (ref)	669 (20.0%)	502 (75.0%)	167 (25.0%)	–	–	–	–
	3	1,004 (30.0%)	753 (75.0%)	251 (25.0%)	0	1	0.80–1.25	0.999
	4	1,071 (32.0%)	803 (75.0%)	268 (25.0%)	0	1	0.80–1.25	0.999
	≥5	598 (17.9%)	449 (75.1%)	149 (24.9%)	0.01	1.01	0.78–1.30	0.95
What is your annual household income?	≤$50,000 (ref)	669 (20.0%)	502 (75.0%)	167 (25.0%)	–	–	–	–
	$51,000–$100,000	1,171 (35.0%)	878 (75.0%)	293 (25.0%)	0	1	0.80–1.25	0.999
	≥$101,000	669 (20.0%)	502 (75.0%)	167 (25.0%)	0	1	0.80–1.25	0.999
	Prefer Not to Disclose	833 (24.9%)	625 (75.0%)	208 (25.0%)	0	1	0.79–1.26	0.999
What is your employment status?	Permanent Employee (ref)	2,005 (60.0%)	1,504 (75.0%)	501 (25.0%)	–	–	–	–
	Contract	1,004 (30.0%)	703 (70.0%)	301 (30.0%)	−0.23	0.79	0.67–0.94	0.007
	Temporary/Visiting	333 (10.0%)	200 (60.1%)	133 (39.9%)	−0.61	0.54	0.42–0.70	<0.001
What is your primary role in the ICU?	Administration (ref)	200 (6.0%)	100 (50.0%)	100 (50.0%)	–	–	–	–
	Nurse	2,005 (60.0%)	1,704 (85.0%)	301 (15.0%)	1.79	6	4.36–8.25	<0.001
	Physician	468 (14.0%)	398 (85.0%)	70 (15.0%)	1.79	6	4.14–8.70	<0.001
	Technician/Other Clinical	669 (20.0%)	468 (70.0%)	201 (30.0%)	0.81	2.25	1.62–3.13	<0.001
How many years have you worked in an ICU?	<1 year (ref)	334 (10.0%)	167 (50.0%)	167 (50.0%)	–	–	–	–
	1–5 years	1,338 (40.0%)	1,137 (85.0%)	201 (15.0%)	1.79	6	4.48–8.04	<0.001
	6–10 years	669 (20.0%)	569 (85.0%)	100 (15.0%)	1.79	6	4.37–8.24	<0.001
	11–20 years	669 (20.0%)	502 (75.0%)	167 (25.0%)	1.1	3	2.22–4.06	<0.001
	>20 years	332 (9.9%)	199 (59.9%)	133 (40.1%)	0.41	1.51	1.09–2.09	0.013
What is your primary shift type?	Day Shift (ref)	1,004 (30.0%)	703 (70.0%)	301 (30.0%)	–	–	–	–
	Night Shift	669 (20.0%)	502 (75.0%)	167 (25.0%)	0.23	1.26	1.02–1.56	0.033
	Rotating Shifts	1,669 (49.9%)	1,252 (75.0%)	417 (25.0%)	0.23	1.26	1.06–1.49	0.008
What is the primary type of ICU you work in?	Medical ICU (ref)	1,171 (35.0%)	878 (75.0%)	293 (25.0%)	–	–	–	–
	Surgical ICU	669 (20.0%)	502 (75.0%)	167 (25.0%)	0	1	0.80–1.25	0.999
	Cardiac ICU	501 (15.0%)	376 (75.0%)	125 (25.0%)	0	1	0.78–1.28	0.999
	Pediatric/Neonatal ICU	334 (10.0%)	251 (75.1%)	83 (24.9%)	0.01	1.01	0.76–1.34	0.947
	Mixed/Other	667 (20.0%)	500 (75.0%)	167 (25.0%)	0	1	0.80–1.25	0.999
Access to workplace mental health resources?	No (ref)	1,671 (50.0%)	919 (55.0%)	752 (45.0%)	-	-	–	–
	Yes	1,338 (40.0%)	1,137 (85.0%)	201 (15.0%)	1.39	4.01	3.35–4.80	<0.001
	Not Sure	333 (10.0%)	167 (50.2%)	166 (49.8%)	−0.2	0.82	0.65–1.03	0.085

### 3.5 Predictors of positive attitudes toward burnout

HCWs aged 25–34 (OR = 4.01, 95% CI 2.92–5.50, *p* < 0.001), 35–44 (OR = 4.01, 95% CI 2.91–5.53, *p* < 0.001), 45–54 (OR = 2.25, 95% CI 1.60–3.17, *p* < 0.001), nurses (OR = 6.00, 95% CI 4.36–8.25, *p* < 0.001), physicians (OR = 6.00, 95% CI 4.14–8.70, *p* < 0.001), technicians (OR = 2.25, 95% CI 1.62–3.13, *p* < 0.001), those with 1–5 (OR = 6.00, 95% CI 4.48–8.04, *p* < 0.001), 6–10 (OR = 6.00, 95% CI 4.37–8.24, *p* < 0.001), or 11–20 years experience (OR = 3.00, 95% CI 2.22–4.06, *p* < 0.001), and with mental health resource access (OR = 4.01, 95% CI 3.35–4.80, *p* < 0.001) had more positive attitudes. Temporary/visiting (OR = 0.54, 95% CI 0.42–0.70, *p* < 0.001) and contract HCWs (OR = 0.79, 95% CI 0.67–0.94, *p* = 0.007) were less likely to have positive attitudes, as shown in Table 6.

**Table 6:** Logistic regression analysis of factors influencing positive attitudes toward burnout among ICU healthcare workers.

Variable	Category	*N* (%)	Positive attitude *N* (%)	Negative attitude *N* (%)	Coefficient (β)	OR	95% CI	*p*-value
What is your age group?	18–24 (ref)	267 (8.0%)	134 (50.2%)	133 (49.8%)	–	–	–	–
	25–34	1,338 (40.0%)	1,071 (80.0%)	267 (20.0%)	1.39	4.01	2.92–5.50	<0.001
	35–44	1,071 (32.0%)	857 (80.0%)	214 (20.0%)	1.39	4.01	2.91–5.53	<0.001
	45–54	468 (14.0%)	328 (70.1%)	140 (29.9%)	0.81	2.25	1.60–3.17	<0.001
	≥55	198 (5.9%)	119 (60.1%)	79 (39.9%)	0.41	1.51	1.01–2.25	0.045
What is your gender?	Female (ref)	2,340 (70.0%)	1,755 (75.0%)	585 (25.0%)	–	–	–	–
	Male	969 (29.0%)	727 (75.0%)	242 (25.0%)	0	1	0.83–1.20	0.999
	Non-binary/Other	33 (1.0%)	25 (75.8%)	8 (24.2%)	0.04	1.04	0.46–2.36	0.927
What is your marital status?	Single (ref)	1,671 (50.0%)	1,254 (75.0%)	417 (25.0%)	–	–	–	–
	Married/Partnered	1,338 (40.0%)	1,004 (75.0%)	334 (25.0%)	0	1	0.84–1.19	0.999
	Divorced/Separated	267 (8.0%)	200 (74.9%)	67 (25.1%)	−0.01	0.99	0.73–1.34	0.947
	Widowed	66 (2.0%)	50 (75.8%)	16 (24.2%)	0.04	1.04	0.59–1.84	0.889
How many members are in your household?	1–2 (ref)	669 (20.0%)	502 (75.0%)	167 (25.0%)	–	–	–	–
	3	1,004 (30.0%)	753 (75.0%)	251 (25.0%)	0	1	0.80–1.25	0.999
	4	1,071 (32.0%)	803 (75.0%)	268 (25.0%)	0	1	0.80–1.25	0.999
	≥5	598 (17.9%)	449 (75.1%)	149 (24.9%)	0.01	1.01	0.78–1.30	0.95
What is your annual household income?	≤$50,000 (ref)	669 (20.0%)	502 (75.0%)	167 (25.0%)	–	–	–	–
	$51,000–$100,000	1,171 (35.0%)	878 (75.0%)	293 (25.0%)	0	1	0.80–1.25	0.999
	≥$101,000	669 (20.0%)	502 (75.0%)	167 (25.0%)	0	1	0.80–1.25	0.999
	Prefer Not to Disclose	833 (24.9%)	625 (75.0%)	208 (25.0%)	0	1	0.79–1.26	0.999
What is your employment status?	Permanent Employee (ref)	2,005 (60.0%)	1,504 (75.0%)	501 (25.0%)	–	–	–	–
	Contract	1,004 (30.0%)	703 (70.0%)	301 (30.0%)	−0.23	0.79	0.67–0.94	0.007
	Temporary/Visiting	333 (10.0%)	200 (60.1%)	133 (39.9%)	−0.61	0.54	0.42–0.70	<0.001
What is your primary role in the ICU?	Administration (ref)	200 (6.0%)	100 (50.0%)	100 (50.0%)	–	–	–	–
	Nurse	2,005 (60.0%)	1,704 (85.0%)	301 (15.0%)	1.79	6	4.36–8.25	<0.001
	Physician	468 (14.0%)	398 (85.0%)	70 (15.0%)	1.79	6	4.14–8.70	<0.001
	Technician/Other Clinical	669 (20.0%)	468 (70.0%)	201 (30.0%)	0.81	2.25	1.62–3.13	<0.001
How many years have you worked in an ICU?	<1 year (ref)	334 (10.0%)	167 (50.0%)	167 (50.0%)	–	–	–	–
	1–5 years	1,338 (40.0%)	1,137 (85.0%)	201 (15.0%)	1.79	6	4.48–8.04	<0.001
	6–10 years	669 (20.0%)	569 (85.0%)	100 (15.0%)	1.79	6	4.37–8.24	<0.001
	11–20 years	669 (20.0%)	502 (75.0%)	167 (25.0%)	1.1	3	2.22–4.06	<0.001
	>20 years	332 (9.9%)	199 (59.9%)	133 (40.1%)	0.41	1.51	1.09–2.09	0.013
What is your primary shift type?	Day Shift (ref)	1,004 (30.0%)	703 (70.0%)	301 (30.0%)	–	–	–	–
	Night Shift	669 (20.0%)	502 (75.0%)	167 (25.0%)	0.23	1.26	1.02–1.56	0.033
	Rotating Shifts	1,669 (49.9%)	1,252 (75.0%)	417 (25.0%)	0.23	1.26	1.06–1.49	0.008
What is your primary type of ICU you work in?	Medical ICU (ref)	1,171 (35.0%)	878 (75.0%)	293 (25.0%)	–	–	–	–
	Surgical ICU	669 (20.0%)	502 (75.0%)	167 (25.0%)	0	1	0.80–1.25	0.999
	Cardiac ICU	501 (15.0%)	376 (75.0%)	125 (25.0%)	0	1	0.78–1.28	0.999
	Pediatric/Neonatal ICU	334 (10.0%)	251 (75.1%)	83 (24.9%)	0.01	1.01	0.76–1.34	0.947
	Mixed/Other	667 (20.0%)	500 (75.0%)	167 (25.0%)	0	1	0.80–1.25	0.999
Access to workplace mental health resources?	No (ref)	1,671 (50.0%)	836 (50.0%)	835 (50.0%)	–	–	–	–
	Yes	1,338 (40.0%)	1,137 (85.0%)	201 (15.0%)	1.39	4.01	3.35–4.80	<0.001
	Not Sure	333 (10.0%)	167 (50.2%)	166 (49.8%)	0.01	1.01	0.80–1.27	0.947

### 3.6 Predictors of effective burnout prevention practices

Healthcare workers aged 25–34 (OR = 4.01, 95% CI 2.92–5.50, *p* < 0.001), 35–44 (OR = 4.01, 95% CI 2.91–5.53, *p* < 0.001), 45–54 (OR = 2.25, 95% CI 1.60–3.17, *p* < 0.001), nurses (OR = 4.01, 95% CI 2.92–5.50, *p* < 0.001), physicians (OR = 3.98, 95% CI 2.75–5.76, *p* < 0.001), technicians (OR = 2.25, 95% CI 1.62–3.13, *p* < 0.001), those with 1–5 (OR = 4.01, 95% CI 2.99–5.38, *p* < 0.001), 6–10 (OR = 4.01, 95% CI 2.92–5.51, *p* < 0.001), or 11–20 years experience (OR = 3.00, 95% CI 2.22–4.06, *p* < 0.001), night shift (OR = 1.26, 95% CI 1.02–1.56, *p* = 0.033) or rotating shift workers (OR = 1.26, 95% CI 1.06–1.49, *p* = 0.008), and those with mental health resource access (OR = 4.01, 95% CI 3.35–4.80, *p* < 0.001) were more engaged in good practices. Temporary/visiting (OR = 0.54, 95% CI 0.42–0.70, *p* < 0.001) and contract HCWs (OR = 0.79, 95% CI 0.67–0.94, *p* = 0.007) were less engaged, as shown in Table 7.

**Table 7:** Logistic regression analysis of factors associated with effective burnout prevention practices among ICU healthcare workers.

Variable	Category	*N* (%)	Good practice *N* (%)	Bad practice *N* (%)	Coefficient (β)	OR	95% CI	*p*-value
What is your age group?	18–24 (ref)	267 (8.0%)	134 (50.2%)	133 (49.8%)	–	–	–	–
	25–34	1,338 (40.0%)	1,071 (80.0%)	267 (20.0%)	1.39	4.01	2.92–5.50	<0.001
	35–44	1,071 (32.0%)	857 (80.0%)	214 (20.0%)	1.39	4.01	2.91–5.53	<0.001
	45–54	468 (14.0%)	327 (69.9%)	141 (30.1%)	0.81	2.25	1.60–3.17	<0.001
	≥55	198 (5.9%)	119 (60.1%)	79 (39.9%)	0.41	1.51	1.01–2.25	0.045
What is your gender?	Female (ref)	2,340 (70.0%)	1,755 (75.0%)	585 (25.0%)	–	–	–	–
	Male	969 (29.0%)	727 (75.0%)	242 (25.0%)	0	1	0.83–1.20	0.999
	Non-binary/Other	33 (1.0%)	25 (75.8%)	8 (24.2%)	0.04	1.04	0.46–2.36	0.927
What is your marital status?	Single (ref)	1,671 (50.0%)	1,254 (75.0%)	417 (25.0%)	–	–	–	–
	Married/Partnered	1,338 (40.0%)	1,004 (75.0%)	334 (25.0%)	0	1	0.84–1.19	0.999
	Divorced/Separated	267 (8.0%)	200 (74.9%)	67 (25.1%)	−0.01	0.99	0.73–1.34	0.947
	Widowed	66 (2.0%)	50 (75.8%)	16 (24.2%)	0.04	1.04	0.59–1.84	0.889
How many members are in your household?	1–2 (ref)	669 (20.0%)	502 (75.0%)	167 (25.0%)	–	–	–	–
	3	1,004 (30.0%)	753 (75.0%)	251 (25.0%)	0	1	0.80–1.25	0.999
	4	1,071 (32.0%)	803 (75.0%)	268 (25.0%)	0	1	0.80–1.25	0.999
	≥5	598 (17.9%)	449 (75.1%)	149 (24.9%)	0.01	1.01	0.78–1.30	0.95
What is your annual household income?	≤$50,000 (ref)	669 (20.0%)	502 (75.0%)	167 (25.0%)	–	–	–	–
	$51,000–$100,000	1,171 (35.0%)	878 (75.0%)	293 (25.0%)	0	1	0.80–1.25	0.999
	≥$101,000	669 (20.0%)	502 (75.0%)	167 (25.0%)	0	1	0.80–1.25	0.999
	Prefer Not to Disclose	833 (24.9%)	625 (75.0%)	208 (25.0%)	0	1	0.79–1.26	0.999
What is your employment status?	Permanent Employee (ref)	2,005 (60.0%)	1,504 (75.0%)	501 (25.0%)	–	–	–	–
	Contract	1,004 (30.0%)	703 (70.0%)	301 (30.0%)	−0.23	0.79	0.67–0.94	0.007
	Temporary/Visiting	333 (10.0%)	200 (60.1%)	133 (39.9%)	−0.61	0.54	0.42–0.70	<0.001
What is your primary role in the ICU?	Administration (ref)	200 (6.0%)	100 (50.0%)	100 (50.0%)	–	–	–	–
	Nurse	2,005 (60.0%)	1,604 (80.0%)	401 (20.0%)	1.39	4.01	2.92–5.50	<0.001
	Physician	468 (14.0%)	374 (79.9%)	94 (20.1%)	1.38	3.98	2.75–5.76	<0.001
	Technician/Other Clinical	669 (20.0%)	468 (70.0%)	201 (30.0%)	0.81	2.25	1.62–3.13	<0.001
How many years have you worked in an ICU?	<1 year (ref)	334 (10.0%)	167 (50.0%)	167 (50.0%)	–	–	–	–
	1–5 years	1,338 (40.0%)	1,071 (80.0%)	267 (20.0%)	1.39	4.01	2.99–5.38	<0.001
	6–10 years	669 (20.0%)	535 (79.9%)	134 (20.1%)	1.39	4.01	2.92–5.51	<0.001
	11–20 years	669 (20.0%)	502 (75.0%)	167 (25.0%)	1.1	3	2.22–4.06	<0.001
	>20 years	332 (9.9%)	199 (59.9%)	133 (40.1%)	0.41	1.51	1.09–2.09	0.013
What is your primary shift type?	Day Shift (ref)	1,004 (30.0%)	703 (70.0%)	301 (30.0%)	–	–	–	–
	Night Shift	669 (20.0%)	502 (75.0%)	167 (25.0%)	0.23	1.26	1.02–1.56	0.033
	Rotating Shifts	1,669 (49.9%)	1,252 (75.0%)	417 (25.0%)	0.23	1.26	1.06–1.49	0.008
What is the primary type of ICU you work in?	Medical ICU (ref)	1,171 (35.0%)	878 (75.0%)	293 (25.0%)	–	–	–	–
	Surgical ICU	669 (20.0%)	502 (75.0%)	167 (25.0%)	0	1	0.80–1.25	0.999
	Cardiac ICU	501 (15.0%)	376 (75.0%)	125 (25.0%)	0	1	0.78–1.28	0.999
	Pediatric/Neonatal ICU	334 (10.0%)	251 (75.1%)	83 (24.9%)	0.01	1.01	0.76–1.34	0.947
	Mixed/Other	667 (20.0%)	500 (75.0%)	167 (25.0%)	0	1	0.80–1.25	0.999
Access to workplace mental health resources?	No (ref)	1,671 (50.0%)	836 (50.0%)	835 (50.0%)	–	–	–	–
	Yes	1,338 (40.0%)	1,071 (80.0%)	267 (20.0%)	1.39	4.01	3.35–4.80	<0.001
	Not Sure	333 (10.0%)	167 (50.2%)	166 (49.8%)	0.01	1.01	0.80–1.27	0.947

## 4 Discussion

This study provides comprehensive insight into burnout KAP among 3,342 HCWs across a regional network of healthcare facilities in Yancheng, Jiangsu, China. The findings extend current literature by identifying specific demographic and occupational predictors of burnout-related KAP, while also revealing gaps in institutional support. Compared to existing global evidence, our data both corroborates and diverges from prior studies, highlighting new perspectives on burnout dynamics in routine, post-pandemic ICU contexts.

The workforce profile revealed a young, predominantly female cohort with a high representation of nurses and mid-career professionals in permanent positions. Access to mental health resources was limited, with 50.0% of HCWs reporting no access, 40.0% confirming access, and 10.0% unsure, consistent with Table 1. The unsure responses may reflect variability in awareness or availability across facilities, underscoring the need for standardized resource dissemination in the regional network. This gap aligns with global concerns regarding insufficient psychological support in critical care settings, particularly in resource-constrained systems. This staffing structure aligns with previous studies indicating that ICU environments globally are often characterized by nurse-dominant, female-majority teams (32, 33). The prevalence of mid-career staff suggests either effective workforce retention or evolving institutional policies in Chinese ICUs that favor continuity and experience accumulation. Interestingly, while the workforce stability appears strong, only 40% of HCWs reported access to mental health resources, echoing widespread concerns regarding the insufficient integration of psychological support within critical care infrastructure (34, 35). Compared to high-income countries where institutional wellness programs are increasingly standardized (36), the persistent resource gap in our setting underscores the disparity in structural support across healthcare systems. This deficit is especially significant given the acknowledged role of organizational interventions in reducing burnout risk and improving resilience among HCWs (37, 38).

Regarding knowledge of burnout, our findings align with previous studies showing that HCWs readily identify workload-related contributors such as long shifts, high patient acuity, and emotional strain (11, 39). However, critical gaps persist in awareness of formal prevention strategies, institutional training initiatives, and actual burnout prevalence. This disconnects between recognition and mitigation knowledge reflects global patterns, particularly in US and European studies, where systemic inertia often delays meaningful intervention (40). Our study adds to this literature by highlighting similar trends in a non-Western ICU context, where stress may be normalized, and institutional solutions underdeveloped. Novel in our findings is the widespread uncertainty surrounding burnout prevalence among peers—a potential result of cultural reticence, insufficient organizational transparency, or the internalization of high stress as normative. This phenomenon has been underreported in prior burnout studies, and its identification here underscores the importance of open discourse and burnout surveillance mechanisms as precursors to systemic reform (12, 41, 42).

Notably, gender, marital status, household size, income, and ICU type were non-significant predictors of KAP outcomes, contrasting with studies linking female sex or specific ICU types (e.g., medical ICUs) to higher burnout risk (43, 44). This discrepancy may reflect contextual factors in the Yancheng healthcare network. Uniformly high workloads across ICU types could minimize differences in burnout vulnerability. Similarly, cultural norms in China, where professional demands often supersede personal circumstances, may reduce the influence of gender or marital status on burnout perceptions. The income’s non-significance may stem from the 24.9% non-disclosure rate, limiting statistical power. These findings suggest that institutional and cultural factors may overshadow demographic influences in this cohort, warranting further exploration in multi-center studies to clarify context-specific drivers.

Attitudinally, HCWs in this study demonstrated strong recognition of burnout’s adverse effects on patient care and staff well-being, a pattern echoed in global evidence (45, 46). The widespread endorsement of institutional support, peer dialog, and structured interventions reaffirms the centrality of systemic responses to burnout prevention (47, 48). However, only moderate self-efficacy in recognizing burnout and considerable uncertainty regarding stigma suggest persisting internal and cultural barriers to effective identification and discussion. These findings align with previous studies identifying stigma and lack of formal training as key deterrents to early recognition (47). Interestingly, participants overwhelmingly rejected the notion that burnout enhances professional identity view that diverges from earlier narratives portraying stress endurance as a marker of professional commitment (49, 50).

Practices reported by ICU staff revealed a strong preference for informal coping mechanisms—such as taking breaks (96.0%), peer discussions (78.9%), and exercise (76.8%)—over formal approaches like counseling (32.0%) or managerial engagement (53.8%). This trend mirrors Western findings where HCWs favor self-initiated strategies due to stigma or access barriers. In the Chinese ICU context, additional cultural and organizational factors likely contribute (50, 51). Mental health stigma, prevalent in China, may deter HCWs from seeking counseling due to fears of professional judgment. Hierarchical hospital structures may discourage managerial discussions, as staff perceive such actions as risking career advancement. Limited visibility and availability of confidential counseling services, with only 40.0% reporting access, further exacerbate underutilization. These barriers suggest the need for culturally sensitive interventions, such as anonymous counseling platforms, leadership-driven stigma reduction campaigns, and mandatory mental health literacy training integrated into staff onboarding across all facilities in the Yancheng network (52–54, 59, 60).

Our regression models identified consistent predictors of favorable KAP outcomes: mid-career age groups, clinical roles (especially nurses and physicians), ICU experience between 1 and 20 years, rotating shift work, and access to mental health resources. These findings support prior research showing that clinical exposure and institutional support are key to effective burnout recognition and prevention (46, 55, 56). Conversely, temporary and contract HCWs were inadequate knowledge, less positive in attitude, and less engaged in preventive practices—a novel and concerning finding. This subgroup likely experiences marginalization in institutional support systems, emphasizing the need for inclusive policy interventions. Notably, variables such as gender, marital status, household size, income, and ICU type did not predict KAP outcomes, contradicting some studies but possibly reflecting contextual stability within our regional multi-institutional cohort.

By applying a KAP framework in a large, post-COVID ICU setting, this study addresses several critical gaps in existing burnout literature and contributes novel evidence to the field. First, it shifts the focus beyond acute crisis settings—such as pandemics—to examine the persistent, systemic stressors contributing to chronic burnout under routine clinical operations. This distinction is vital for designing sustainable interventions. Second, it links behavioral burnout outcomes with granular sociodemographic and occupational predictors, allowing for precision in intervention targeting—an approach often lacking in broader prevalence-focused studies. Third, it brings to light disparities in knowledge, attitudes, and preventive behaviors among non-permanent HCWs, a group routinely excluded from institutional wellness programs despite elevated vulnerability. This inclusion enhances the equity and inclusivity of burnout research. Lastly, the large sample size and multi-domain KAP design offer a robust methodological contribution, making this study one of the few to comprehensively assess burnout cognition and behavior at scale within a high-risk ICU environment in a non-Western context.

The regional focus on Yancheng, Jiangsu, limits the generalizability of findings to other healthcare systems, particularly those with differing organizational cultures or resource availability. Chinese ICUs often operate within hierarchical structures, which may discourage formal support-seeking due to perceived professional risks or mental health stigma, a factor less prominent in Western settings with standardized wellness programs. Resource constraints in China’s public hospitals may exacerbate gaps in mental health support compared to high-income countries. These cultural and systemic factors likely influenced the preference for informal coping and low counseling uptake observed, underscoring the need for context-specific interventions. Multi-center studies spanning diverse regions are needed to enhance the applicability of findings.

The multicenter design of this study was confined to 10 hospitals within a single city, Yancheng, Jiangsu. Consequently, the findings are context-specific and may not be readily generalizable to ICUs in other Chinese provinces or to healthcare systems with different organizational structures and resource availability. The cross-sectional nature of the study design inherently precludes causal inferences and an analysis of temporal trends in burnout. Although a high response rate of 74.3% was achieved, the potential for selection bias cannot be fully dismissed, as non-respondents might systematically differ from participants (e.g., in terms of workload, motivation, or burnout severity). The exclusive use of self-reported data for KAP is a limitation due to potential recall and social-desirability biases. Lastly, the evaluation of mental health resource accessibility relied entirely on participant self-report, lacking external validation of the quality or scope of these services.

## 5 Conclusion

This study reveals that ICU staff in a post-pandemic, multi-institutional context in Yancheng, Jiangsu, experience substantial burnout, with knowledge, attitudes, and practices influenced by clinical role, ICU experience, shift schedules, and institutional support availability. Temporary and contract staff demonstrated lower KAP scores, highlighting structural vulnerabilities. The predominance of informal coping strategies points to cultural and organizational barriers to formal support engagement. These findings, while robust within the regional context, underscore the need to standardize mental health support, enhance burnout training, and integrate non-permanent staff into wellness initiatives. The KAP framework provides a scalable tool for behavioral monitoring, but broader studies are needed to generalize findings across diverse healthcare systems.

## Data Availability

The raw data supporting the conclusions of this article will be made available by the authors, without undue reservation.
